# Predictive Value of the Early Spontaneous Movements for Preterm Infants’ Neurodevelopmental Outcome at 12 Months’ Corrected Age

**DOI:** 10.1002/brb3.70562

**Published:** 2025-05-19

**Authors:** Ayse Yildiz, Ramazan Yildiz, Umut Apaydin, Pelin Atalan Efkere, Rabia Zorlular, Bulent Elbasan

**Affiliations:** ^1^ Department of Physical Therapy and Rehabilitation, Faculty of Health Sciences Erzurum Technical University Erzurum Turkey; ^2^ Department of Physiotherapy and Rehabilitation, Faculty of Health Sciences Karadeniz Technical University Trabzon Turkey; ^3^ Department of Physical Therapy and Rehabilitation, Faculty of Health Sciences Gazi University Ankara Turkey; ^4^ Department of Physical Therapy and Rehabilitation, Bor Faculty of Health Sciences Nigde Omer Halisdemir University Niğde Turkey

**Keywords:** neurodevelopmental outcome, preterm infant, sensory processing

## Abstract

**Purpose:**

Motor, cognitive, behavioral, and sensory problems may be seen in preterm infants. Early spontaneous movements' role in these developmental areas has yet to be sufficiently investigated. This study aimed to evaluate the effectiveness of the Motor Optimality Score‐Revised (MOS‐R) in predicting motor, cognitive, language, and sensory developmental outcomes in premature infants.

**Methods:**

Forty preterm children were examined. Early spontaneous movements were evaluated using the General Movement Assessment (GMA), which detects the MOS‐R. Infants' language, cognitive, and motor development were assessed using the Bayley Scales of Infant and Toddler Development, Third Edition (Bayley‐III). Sensory development was evaluated using the Test of Sensory Functions in Infants (TSFI).

**Results:**

The mean score for the MOS‐R was 23.13 ± 4.6. Most infants (*n* = 31, 77.5%) showed typical fidgety movements. The sensitivity of the MOS‐R for determining motor, cognitive, and language development at 12 corrected months was 86.7%, 80%, and 82%, respectively, and the specificity was 71.4%, 100%, and 100%, respectively. MOS‐R scores did not predict sensory development outcomes (*p* > 0.05)

**Conclusions:**

At three months, the MOS‐R demonstrated high sensitivity and specificity in detecting motor, cognitive, and language functions in preterm infants at 12 months corrected age.

## Graphical abstract

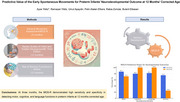

## 1 Introduction

Preterm infants are more likely to face motor, behavioral, and cognitive problems compared to their term peers (Johnson 2007). Although neonatal intensive care unit (NICU) advancements have improved survival rates, preterm infants remain vulnerable to neurodevelopmental disorders (VandenBerg 2007). Early diagnosis is crucial for implementing interventions that support development, minimize complications from brain injury, and improve long‐term outcomes (Constantinou et al. 2007; Craciunoiu and Holsti 2017).

Clinical evaluation of newborns plays a key role in predicting later neurological disorders. Several assessment batteries have been developed for this purpose, with studies examining their predictive accuracy. The Neonatal Behavioral Assessment Scale (NBAS) evaluates individual differences in neonatal behavior and has been identified as a predictor of developmental disabilities in longitudinal studies (Brazelton and Nugent 1995; Ohgi et al. 2003). Similarly, the NICU Network Neurobehavioral Scale (NNNS), which integrates neurological and behavioral examination, has been linked to adverse cognitive, motor, and behavioral outcomes in high‐risk preterm infants at two years of age, demonstrating substantial long‐term predictive value (El‐Dib et al. 2012; Stephens et al. 2010; Mcgowan et al. 2022). The Rapid Neonatal Neurobehavioral Assessment Procedure (RNNAP) assesses the integrity and organization of the sensory‐motor system, with studies indicating that high‐risk infants with typical early performance tend to develop normally. In contrast, persistent abnormal movements predict poorer outcomes (Gardner et al. 2006). The Hammersmith Neonatal Neurological Examination (HNNE), a widely used neurological assessment in preterm infants, has shown moderate predictive accuracy for motor outcomes at 12 months of corrected age (Howard et al. 2023). While these tools provide valuable insights, additional assessments, such as the General Movement Assessment (GMA), have been developed to enhance early detection of neurological deficits in preterm infants before they reach term age (Hfr 2001; Ferrari et al. 2002).

General movements (GMs) are complex, spontaneous movements involving coordinated activity of the arms, legs, neck, and trunk. These movements emerge early in fetal life and continue postnatally. Up to 40 weeks of gestation are classified as Writhing Movements, followed by Fidgety Movements from 9 to 20 weeks post‐term (Einspieler and Prechtl 2005). Qualitative assessment of GMs during the fidgety movement period is a prognostic tool for identifying infants at risk of neurodevelopmental disabilities (Burger and Louw 2009). Studies report that GMA during fidgety movements has high sensitivity (80%–100%) but lower specificity (60%–68.8%) for predicting motor outcomes at 12 and 24 months (Robinson et al. 2021). While GMA at one month of age has shown high sensitivity (>80%) for predicting motor outcomes at one year, its overall predictive accuracy remains limited (Spittle et al. 2009). Although the general GMA is an important tool for evaluating GMs, the Motor Optimality Score‐Revised (MOS‐R) was developed to provide a more detailed and holistic assessment of early motor development (Bertoncelli et al. 2024, Paris et al. 2023).

MOS‐R is a structured clinical tool that evaluates an infant's movement, posture, and age‐appropriate development (Einspieler et al. 2019). While NNNS and HNNE evaluate broader neurobehavioral and neurological aspects, MOS‐R provides a detailed perspective on motor optimality in premature infants, focusing specifically on early motor development. Notably, it can identify infants with apparently normal fidgety movements who may still be at risk for neurodevelopmental difficulties, offering an advantage in early screening (Kwong et al. 2022). While most research on MOS‐R has focused on predicting cerebral palsy (CP) and motor impairments, emerging evidence suggests that lower MOS‐R scores may also indicate other developmental challenges (Kwong et al. 2022). Studies have linked reduced MOS‐R scores to motor and language dysfunction in toddlers and later learning difficulties (Crowle et al. 2023). Studies on MOS‐R and sensory processing skills are minimal, with only one study reporting that fidgety movements were associated with typical/atypical movement processing in preterm toddlers (Yardımcı‐Lokmanoğlu et al. 2021).

Sensory processing is critical for infants' ability to engage with their environment, influencing motor planning, cognitive skills, and social‐emotional development. Preterm infants are particularly vulnerable to sensory processing difficulties, which can manifest as atypical responses to stimuli, impaired motor coordination, or later learning deficits. Given the fundamental role of sensory processing in early development, investigating whether early assessment tools can predict sensory problems is essential (Eeles et al. 2013; Ryckman et al. 2017).

Although MOS‐R is well established for predicting CP and motor disorders, its potential to predict broader neurodevelopmental outcomes, particularly sensory processing, remains largely unexplored. Since sensory processing is integral to motor learning, cognition, and social‐emotional development, early identification of difficulties could enhance intervention strategies. This study aims to fill this gap by evaluating whether the MOS‐R in 12‐month‐old premature infants can predict both motor outcomes and sensory, cognitive, and language difficulties.

## 2 Methods

### 2.1 Participants

This study was conducted in the Department of Pediatric Rehabilitation, Faculty of Health Sciences, Gazi University. The study included infants born before 37 weeks of gestation who spent at least 15 days in the NICU. Families who consented to participate signed an informed consent form. Exclusion criteria included infants with congenital anomalies, metabolic, or genetic disorders, or those requiring ventilator support. Video recordings were made at the corrected age of 3 months for 45 infants meeting the inclusion criteria. Five infants were withdrawn during the one‐year follow‐up, leaving 40 preterm infants who reached the corrected age of 12 months. The Gazi University Ethics Committee approved all research procedures. (Number: 77082166–604.01‐920750).

Sample size calculations were performed using G*Power (Version 3.1.9.7, University of Düsseldorf, Germany) (Faul et al. 2007). A biserial correlation model was applied to examine the primary correlation between the MOS‐R and Bayley‐III, with an expected correlation coefficient of 0.40, α = 0.05, and power of 1‐β = 0.80. Based on these parameters and reference values from Spittle et al., a minimum sample size of 34 participants was required. (Spittle et al. 2013).

### 2.2 Procedure

GM of infants aged three months corrected age was recorded by a physiotherapist with ten years of experience in pediatrics. A 3–5‐min video recording was captured of infants lying in a supine position while being active and alert (Einspieler et al. 1997). Two assessors, certified by the GM Trust for global and detailed assessment, independently assessed the video recordings. In the event of a disagreement, the responsible author informed the assessors, who then viewed the videos together and reached a consensus. Participants were evaluated using the Bayley scales of infant and toddler development, third edition (Bayley‐III) and test of sensory functions in infants (TSFI) at 12 months corrected age by an assessor blinded to the MOS‐R score and the children's perinatal history.

### 2.3 Measurements

#### 2.3.1 General Movements Assessment (GMA)

The GMA was used to determine the presence of fidgety movements in infants, and the MOS‐R via video recordings were recorded retrospectively at three months of corrected age. Infants have spontaneous GM from birth to 20 weeks post‐term (Örtqvist et al. 2021). The fidgety movements were recorded as present/absent. The MOS‐R, a detailed GM evaluation, scores between 5 (minimum) and 28 (optimal performance). Scores between 25 and 28 are considered optimal, while scores below 25 indicate decreased performance. All assessments followed the standard GM observation protocol, with infants partially dressed and positioned to allow free movement in a supine position (Einspieler et al. 1997).

#### 2.3.2 The Bayley Scales of Infant and Toddler Development, Third Edition (Bayley‐III)

Bayley‐III scale for infants' cognitive, language, and motor development. At a time when the infant was calm and happy, the evaluation was made by a certified physiotherapist who was trained to apply the test in a clinic where appropriate environmental conditions were provided. Bayley‐III is a scale that evaluates five developmental areas in children aged 1–42 months: cognitive development, language development, motor development, social‐emotional development, and adaptive behavior. The Bayley‐III does not offer an overall total score; instead, it provides raw and scaled scores for each domain, composite scores, and percentile ranks for each scale. All items on the scale are scored as 0 or 1, and a raw score is obtained. These raw scores are converted into scale scores and then composite scores (mean: 100, standard deviation: 15) [166].

#### 2.3.3 Test of Sensory Functions in Infants (TSFI)

The TSFI is a tool that investigates the sensory functioning in infants aged 4–18 months. The tool determines if an infant has sensory processing deficits and the type of deficits they have. It includes 24 items that each can be scored, as well as five subdomains, including reactivity to tactile deep pressure, adaptive motor responses, visual‐tactile integration, oculomotor control, and vestibular stimulation. A maximum 49‐point score can be obtained from the scale, and the higher scores represent better sensory processing functioning (DeGangi and Greenspan 1989). In this study, the TSFI was administered by a physiotherapist with 10 years of experience in pediatric rehabilitation.

### 2.4 Statistical Analysis

Data was analyzed using Version 26.0 of the SPSS program (IBM SPSS Statistics, Chicago, IL, USA). Both analytical techniques, such as the Shapiro–Wilk test, and visual methods, like histograms, were employed to assess normality. For normally distributed data, descriptive statistics are reported as the mean and standard deviation; for non‐normally distributed data, they are reported as the median and interquartile range. Descriptive statistics for qualitative variables are presented as percentages and frequencies. The relationships between the MOS‐R optimality category and the Bayley‐III outcome level were examined using chi‐squared association tests. The sensitivity, specificity, and corresponding 95% confidence intervals for 1‐year outcomes predicted by total MOS‐R scores were calculated to evaluate predictive validity. The area under the ROC curve was computed to identify the MOS‐R total score cut‐off values with the highest predictive accuracy. Spearman correlation coefficient (*r*) was used to evaluate the relationship between MOS‐R and TSFI subparameters. The *r* values were interpreted as follows: 0.00–0.19, very weak; 0.20–0.39, weak; 0.40–0.59, moderate; 0.60–0.79, strong; and 0.80–1.00, very strong correlation (Mukaka 2012). Statistical significance was established at *p* < 0.05.

## 3 Results

The median gestational age of the infants included in the study was 34 weeks, median birth weight was 2550 g, and NICU stay was 17 days. Nineteen (47.5%) infants were male and 21 (52.5%) were female (Table 1).

**TABLE 1:** Demographic characteristics of infants.

Demographic characteristics (*n*: 40)		Median (IQR)	
Gestational age (week)		34 (29–38)	
Birth weight (gr)		2550 (1200–3150)	
Length of NICU stay		17 (4–60)	
Maternal age (years)		31 (27.5–32.75)	
Paternal age (years)		35 (30–36)	
Demographic characteristics (*n*: 40)		***n***	**(%)**
Gender	Male	19	47.5
	Female	21	52.5
Type of birth	Normal	10	25
	Cesarean	30	75
Oxygen support	None	10	25
	Less than 24 h	8	20
	1–7 days	10	25
	More than seven days	12	30
Mother's education	Secondary school	2	5
	High school	17	42.5
	Bachelor degree	20	50
	MSc, PhD	1	2.5

Abbreviations: MSc, Master of Science; NICU, neonatal intensive care unit; PhD, Doctor of Philosophy.

The mean score for the MOS‐R was 23.13 ± 4.6 (Table 2). Most infants (*n* = 31, 77.5%) showed typical fidgety movements. Twenty‐nine infants (72.5%) had MOS‐R (25–28) scores within the optimal range, while two infants (5%) scored between 20 and 24, seven infants (17.5%) scored between 9 and 19, and two infants (5%) scored between 5 and 8. Nine infants (22.5%) were determined to be at risk of benefiting from referral to early intervention based on the MOS‐R categorization (MOS‐R < 20).

**TABLE 2:** Motor Optimality Score‐Revised results (*n*: 40).

Total MOS‐R	Median (IQR) 26 (22–28)
Fidgety movements	***n* (%)**
Normal	31 (77.5)
Abnormal	2 (5)
Absent/sporadic	7 (17.5)
Observed movement patterns	***n* (%)**
Normal > Abnormal	36 (90)
Normal = Abnormal	4 (10)
Normal < Abnormal	0 (0)
Age‐adequate movement repertoire	***n* (%)**
Present	22 (55)
Reduced	1 (2.5)
Absent	17 42.5)
Observed postural patterns	***n* (%)**
Normal > Atypical	31 (77.5)
Normal = Atypical	6 (15)
Normal < Atypical	3 (7.5)
Movement character	***n* (%)**
Smooth and fluent	31 (77.5)
Abnormal, not cramped synchronized	7 (17.5)
Cramped synchronized	2 (5)

Abbreviation: MOS‐R, Motor Optimality Score‐Revised.

Regarding movement patterns, 36 infants (90%) exhibited predominantly standard movement patterns, although half of the cohort (*n* = 18, 45%) demonstrated less or no age‐appropriate repertoire. Thirty‐one infants were identified with typical movement characteristics, while only 22.5% exhibited abnormal or cramped synchronized movements (Table 2).

Based on each developmental domain, the classification of infants into MOS‐R optimality categories at 3 months and their developmental outcomes at 12 months are summarized in Table 3.

**TABLE 3:** MOS‐R optimality category and classification of each developmental domain category at 12 months corrected age.

	Bayley‐III Motor		*p*	Bayley‐III Cognitive		*p*	Bayley‐III Language		*p*	TSFI		*p*
**MOS‐R**	<85	≥ 85	**0.002**	<85	≥ 85	**0.010**	<85	≥ 85	**<0.001**	<40	≥ 40	0.620
≥ 24	2	27		0	29		0	29		6	23	
										%15	%57.5	
							%0	%72.5				
				%0	%72.5							
	%5	%67.5										
24–20	0	2		0	2		0	2		0	2	
										%0	%5	
							%0	%5				
	%0	%5		%0	%5							
19–9	3	4		1	6		1	6		1	6	
										%2.5	%15	
							%2.5	%15				
				%2.5	%15							
	%7.5	%10										
8–5	2	0		1	1		2	0		1	1	
										%2.5	%2.5	
							%5	%0				
				%2.5	%2.5							
	%5	%0										

Abbreviations: Bayley‐III, Bayley Scales of Infant and Toddler Development‐III; MOS‐R, Motor Optimality Score‐Revised; P, Pearson chi‐square test; TSFI, Test of Sensory Functions in Infants.

Total MOS‐R showed moderate positive correlations with Visual Tactile Integration (*r* = 0.460, *p* = 0.004), Adaptive Motor Function (*p* <0.001, *r* = 0.550), Reactivity to Vestibular Stimulation (*p* = 0.008, *r* = 0.429) and TSFI total score (*p* = 0.002, *r* = 0.475) (Table 4).

**TABLE 4:** Correlations between Motor Optimality Score and Test of Sensory Functions in Infants.

Spearman *r*, *p* value	Reactivity to Tactile Deep Pressure	Visual Tactile Integration	Adaptive Motor Function	Ocular Motor Control	Reactivity to Vestibular Stimulation	Total TSFI score
Fidgety movements	*r*: 0.005	*r*: 0.265	***r*: 0.328**	***r*: 0.484**	*r*: 0.204	*r*: 0.186
	*p*: 0.976	*p*: 0.114	***p*: 0.048**	***p*: 0.002**	*p*: 0.225	*p*: 0.250
Observed movement patterns	*r*: −0.110	***r*: 0.364**	*r*: 0.137	*r*: 0.302	*r*: 0.190	*r*: 0.194
	*p*: 0.516	***p*: 0.027**	*p*: 0.420	*p*: 0.070	*p*: 0.261	*p*: 0.231
Age‐adequate movement repertoire	***r*: 0.433**	***r*: 0.498**	***r*: 0.557**	*r*: 0.269	***r*: 0.484**	***r*: 0.537**
	***p*: 0.007**	***p*: 0.002**	***p* < 0.001**	*p*: 0.107	***p*: 0.002**	***p* < 0.001**
Observed postural patterns	*r*: −0.195	*r*: 0.170	*r*: 0.236	*r*: 0.187	*r*: 0.130	*r*: 0.145
	*p*: 0.248	*p*: 0.313	*p*: 0.159	*p*: 0.269	*p*: 0.443	*p*: 0.371
Movement character	*r*: −0.126	*r*: 0.297	*r*: 0.307	***r*: 0.438**	*r*: 0.166	*r*: 0.151
	*p*: 0.458	*p*: 0.074	*p*: 0.064	***p*: 0.007**	*p*: 0.325	*p*: 0.354
Total MOS‐R	*r*: 0.290	***r*: 0.460**	***r*: 0.550**	***r*: 0.370**	***r*: 0.429**	***r*: 0.475**
	*p*: 0.081	***p*: 0.004**	***p* < 0.001**	***p*: 0.024**	***p*: 0.008**	***p*: 0.002**

Abbreviations: MOS‐R, Motor Optimality Score‐Revised; TSFI, Test of Sensory Functions in Infants.

ROC curve analysis evaluated the predictive validity of MOS‐R scores (Table 5). When analyzing the data to predict delays in the Bayley‐III motor composite score, a MOS‐R total score of less than 21.5 indicated a postponement with a sensitivity of 0.86 (PPV 0.92) and specificity of 0.71 (NPV 0.55) and an area under the ROC curve (AUC) of 0.83. Similar results were valid for language and cognitive composite scores. However, the NPV was low for these areas. The predictive values of MOS‐R scores for TSFI score were insignificant (Table 5).

**TABLE 5:** Predictive values of the MOS‐R, according to Bayley‐III score and TSFI score.

Initial assessment	Outcome assessment	Sensitivity	Specificity	PPV	NPV	AUC	% 95 confidence intervals	*p*
GMs Total MOS‐R Score (Cut‐off 21.5 points)	**Bayley‐III motor score** (cut‐off 85 points)	86.7	71.4	92.9	55.6	0.883	0.770–0.997	**0.002**
	**Bayley‐III cognitive score** (cut‐off 85 points)	80	100	100	22.2	0.950	0.858–1	**0.034**
	**Bayley‐III language score** (cut‐off 85 points)	82	100	100	33.3	0.975	0.921–1	**0.007**
	**TSFI score** (cut‐off 40 points)	75	25	78	22.2	0.728	0.566–0.80	0.051

Abbreviations: AUC, Area Under Curve; Bayley‐III, Bayley Scales of Infant and Toddler Development‐III; MOS‐R, Motor Optimality Score‐Revised; NPV, Negative Predictive Value; PPV, Positive Predictive Value; TSFI, Test of Sensory Functions in Infants.

## 4 Discussion

This study examined the ability of the MOS‐R to predict motor, language, cognitive, and sensory developmental outcomes in preterm infants at 12 months corrected age. The results showed that MOS‐R at three months demonstrated high sensitivity and specificity in predicting motor, cognitive, and language development at 12 months.

GMA has long been recognized as a sensitive method for predicting infant neurodevelopmental disorders. Specifically, fidgety movements during the fidgety period have been shown to have excellent sensitivity and specificity in predicting CP (Cioni et al. 1997). In addition, the MOS‐R is a reliable predictor of CP when identifying infants more likely to experience difficulties in early childhood (Einspieler et al. 2019; Kwong et al. 2022; Crowle et al. 2023). However, the sensitivity and specificity of early spontaneous movement quality in predicting motor development vary across studies. For example, Sustersic et al. reported high sensitivity (100%) but low specificity (24%) for GMA at three months in predicting motor impairments (Sustersic et al. 2012), while Kodric et al. found a sensitivity of 61% and specificity of 46% (Kodric et al. 2010). Spittle et al. reported a sensitivity of 70% and specificity of 85% for GMA at three months in predicting motor impairment at two years (Spittle et al. 2013). In the present study, MOS‐R showed high specificity and PPV in predicting motor impairment at 12 months. These findings suggest that those with good MOS‐R values ​​at three months of corrected age may have good motor development. However, given that the morbidity of the cohort may affect the positive predictive value, caution should be exercised in generalizing these results.

The persistent absence or abnormal presence of fidgety movements has been associated with the development of clinical conditions such as mental retardation and CP, with a reliability of 93%–96% (Einspieler et al. 1997; Prechtl et al. 1997; Hadders‐Algra 1996; Cioni et al. 1997). A detailed analysis of GM quality in premature infants at 3 to 5 months post‐term age was reported to predict intelligence at 7 to 10 years of age (Fjørtoft et al. 2013; Grunewaldt et al. 2014). Kodric et al. found that GMA had 83% sensitivity and 55% specificity in predicting cognitive impairment at three months (Kodric et al. 2010). At three months, Spittle et al. found a relationship between abnormal GMs and language and cognitive development (Spittle et al. 2013). The present study found that the MOS‐R results of preterm infants showed high sensitivity and specificity in predicting cognitive and language development at one year. This may be due to the strong association between GMs and white matter abnormalities. Recently, a clear association was found between abnormal GMs 1 to 3 months after term and cerebral white matter abnormalities on MRI scans at term age (Spittle et al. 2008). Cerebral white matter damage, especially, has been linked to cognitive dysfunction in preterm infants at school age, often occurring without significant motor deficits (Woodward et al. 2006; Edgin et al. 2008). Thus, it is unsurprising that GM quality shows high sensitivity and specificity for predicting cognitive and language development.

While GMA is primarily used to predict motor disorders, particularly CP, recent research has also explored the relationship between GMs and cognitive and language development in preterm infants. However, few studies have examined the relationship between GMs and sensory development. Yardımcı‐Lokmanoğlu et al. examined the relationship between GMA and sensory processing skills in preterm toddlers. They found no relationship between MOS and sensory development (Yardımcı‐Lokmanoğlu et al. 2023). In another study, Yardımcı‐Lokmanoğlu et al. found that preterm infants who displayed abnormal restless movements between 3 and 5 months showed more atypical movement sensory processing and touch sensory processing skills between 24 and 35 months. They also reported that fidgety movements were related to movement‐processing skills (Yardımcı‐Lokmanoğlu et al. 2021). These studies relied on parent reports to assess sensory processing, which may have introduced biases. In contrast, the current study employed the more objective TSFI to mitigate potential sociocultural differences. The study found that the total MOS‐R score at 3 months was significantly correlated with sensory processing skills at 12 months but did not predict sensory development at 12 months. These findings suggest that early motor functions may be linked to sensory processing in the long term but that the MOS‐R may not be sufficient to predict sensory processing skills directly. In other words, having good motor optimality at 3 months may provide an advantage for sensory processing. Still, factors such as environmental stimuli, early experiences, parental interaction, and neuroplasticity also play a significant role in shaping sensory functions. Therefore, it may be difficult to predict with certainty how sensory processing skills will develop in an infant with good motor optimality. Sensory processing processes can change over time, and early motor assessments such as the MOS‐R may not adequately capture the more complex aspects of the sensory system.

## 5 Limitations

A limitation of this study is the heterogeneous nature of the sample, particularly concerning gestational age and additional perinatal risk factors. Although the sample size was sufficient to predict motor development in our study, this was not the case for other parameters. Also, the sample size does not enable the exploration of the fundamental effects of prematurity severity, which has been shown previously to be highly relevant for GMA and the dependent variables. Thus, future studies should consider being repeated in a larger sample size and grouping infants by gestational age to minimize variability. In addition, the assessment at 12 months may not be sufficient to fully assess the long‐term neurodevelopmental outcomes of at‐risk infants. The short observation period in this study further limits the ability to conclude developmental trajectories. Therefore, more extended follow‐up studies with larger sample sizes are needed to provide more comprehensive insights.

## 6 Conclusion

The MOS‐R strongly predicts neurodevelopmental outcomes in 12‐month‐old preterm infants, demonstrating high sensitivity and specificity for motor, cognitive, and language development. However, no test is 100% accurate due to the influence of social, environmental, and biological factors on development. Clinicians and researchers should consider that abnormal movements are linked to motor deficits and challenges in other developmental domains. Further research is needed to compare the validity of GMA and explore its correlation with long‐term developmental outcomes.

## Author Contributions

**Ayse Yildiz**: methodology, writing–original draft, formal analysis. **Ramazan Yildiz**: methodology, writing–original draft, formal analysis, data curation. **Umut Apaydin**: data curation, writing–original draft, methodology. **Pelin Atalan Efkere**: data curation. **Rabia Zorlular**: data curation. **Bulent Elbasan**: supervision.

## Ethics Statement

The Gazi University Clinical Research Ethics Committee received permission to conduct the study (Number: 14574941‐302.08.01‐). Parents were asked to sign a consent form to allow their infants to participate in the study.

## Consent

The authors have nothing to report.

## Conflicts of Interest

The authors declare no conflicts of interest.

### Peer Review

The peer review history for this article is available at https://publons.com/publon/10.1002/brb3.70562.

## Data Availability

The data that support the findings of this study are available on request from the corresponding author. The data are not publicly available due to privacy or ethical restrictions.
